# Alcohol Preference Impacts Multi-Organ Transcriptome in MetALD

**DOI:** 10.3390/genes16101121

**Published:** 2025-09-23

**Authors:** Saumya Sikhwal, Tyler C. Gripshover, Rui S. Treves, Josiah E. Hardesty

**Affiliations:** 1 Department of Pharmacology and Toxicology, University of Louisville School of Medicine, Louisville, KY 40202, USA; saumya.sikhwal@louisville.edu (S.S.); tygripshover72@gmail.com (T.C.G.); rui.treves@louisville.edu (R.S.T.); 2 Department of Psychological and Brain Sciences, University of Louisville, Louisville, KY 40202, USA; 3 University of Louisville Alcohol Research Center, University of Louisville School of Medicine, Louisville, KY 40202, USA

**Keywords:** AUD, MetALD, transcriptome, ALD, HFD

## Abstract

Background/Objectives: Alcohol use disorder (AUD) is a major public health issue with rising global occurrence and metabolic consequences. Modeling the addictive behaviors associated with AUD remains inadequate and elusive. Even more so, models that are representative of AUD in concert with excessive caloric intake are limited. Some consequences of chronic alcohol use overlap with the metabolic phenotype of hypercaloric diets. Recently characterized metabolic dysfunction-associated steatotic liver disease with increased alcohol intake (MetALD) helps to differentiate these conditions. This study aims to investigate metabolic phenotypes and gene expression alterations in MetALD mice that are grouped by alcohol preference based on blood phosphatidylethanol levels and alcohol consumption. Methods: Mice were fed high-fat and chow diets, with water and 10% EtOH, for 13 weeks. mRNA sequencing was performed across multiple tissues including brain, liver, skeletal muscle, ileum, and white adipose tissue, and gut microbiome diversity was evaluated via 16S sequencing. Results: Key findings included reduced glucagon in alcohol-preferring mice with no significant differences in dyslipidemia and hepatic steatosis. Additionally, we observed reduced gut microbiome diversity and Wnt signaling with elevated acute-phase response genes in ileum tissue. Reduced Wnt and Hippo signaling in the brain and liver, respectively, was also revealed. Other gene ontologies discovered included increased neural inflammation and adipose mitochondrial translation. *Nek3, Ntf3, Cux1*, and *Irf6* expression changes were shared across at least three tissues and may be potential biomarkers of alcohol addiction. Conclusions: This novel model assists future intervention research in the characterization of MetALD and identifies potential biomarkers of alcohol preference.

## 1. Introduction

Alcohol is the most widely consumed recreational substance worldwide [1]. The World Health Organization estimates that in 2016, about 2.3 billion people aged 15 or older had consumed alcohol in the previous 12 months [2]. Chronic alcohol consumption can lead to the development of alcohol use disorder (AUD), which is associated with considerable disability, as it is the third-most disabling disease category in high-income countries [2]. According to the National Institutes of Health (NIH), AUD is defined as a medical condition characterized by the inability to control alcohol use despite adverse consequences [3]. AUD is a complex neuropsychiatric condition that combines behavioral, neurobiological, and psychosocial aspects, with 9.7% of the U.S. population aged 12 or older meeting the AUD diagnostic criteria [4]. The United States alone spends USD 224 billion annually to combat AUD and mortality has increased roughly 29% from 2016–2017 to 2020–2021 [5]. This increase is likely associated with the COVID-19 pandemic where alcohol-related complications rose during the months of quarantine [6]. While AUD is a growing public health crisis, much of the existing research focuses on organ pathology and associated metabolic endpoints. More work is required to understand the complex interaction of AUD’s neurophysiology and its effects on the whole body. In addition, most AUD studies do not also take into consideration the effect of diet and nutrition. This is a significant and impactful modifying factor due to the hypercaloric environment of Western society. Recently, multiple professional research societies have characterized and coined the pathology of metabolic dysfunction-associated steatotic liver disease with increased alcohol intake (MetALD) [7]. This clarification better identifies and categorizes pathologies that comprise both high-fat or non-nutritious diets with alcohol consumption patterns. While there have been some studies that have identified mechanisms of MetALD and AUD, no one has attempted to identify biomarkers that contribute to alcohol preference in a MetALD model [8,9].

The goal of this study is to investigate metabolic and gene expression alterations in MetALD mice in the context of AUD. Specifically, this study was performed with male C57Bl/6J mice to inspect the onset of gene alterations in multiple tissues that may interact with alcohol preference. Mice were provided *ad libitum* access to a high-fat diet, standard rodent chow, water, and 10% EtOH and later separated based on alcohol preference status via blood phosphatidylethanol levels [10] and alcohol drinking patterns. Our objective was to identify potential metabolic and gene biomarkers that distinguish alcohol-preferring and alcohol non-preferring mice. We analyzed gut microbial diversity and mRNA sequencing data for matching whole liver, brain, skeletal muscle, white adipose tissue, and ileum tissues. We hypothesize that alcohol-preferring mice will have a more severe metabolic phenotype and have induced genes affiliated with previously established markers of AUD or chronic alcohol consumption.

## 2. Materials and Methods

### 2.1. Animal Study

**Figure 1:**
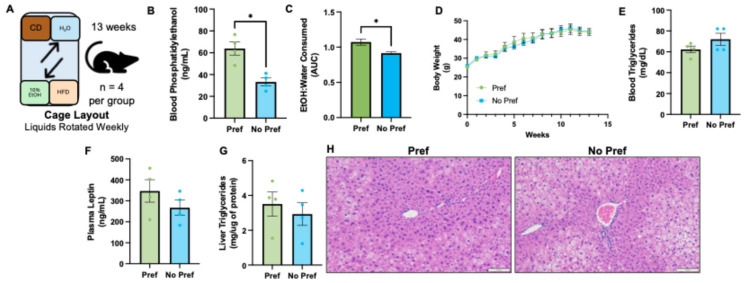
Alcohol-preferring and non-preferring mice have similar liver lipid changes. (**A**) Diagram of diets and liquids layout in each cage of mice, where liquids were rotated weekly. (**B**) Blood phosphatidylethanol (ng/mL). (**C**) EtOH consumed relative to water for final 4 weeks of study presented as area under the curve. (**D**) Mouse body weights (g), weighed weekly over the course of study. (**E**) Blood triglycerides (mg/dL). (**F**) Plasma leptin levels (ng/mL). (**G**) Liver triglycerides normalized to total liver protein (mg/µg). (**H**) Representative H&E staining of the liver for each group (scale bar set to 100 um). An unpaired Student’s *t*-test was used to statistically compare our two groups where *p* < 0.05 was considered statistically significant and denoted by a *.

For this study, 10–12-week-old male C57BL/6J mice were purchased from Jackson Laboratory. Experimental animals were housed in a temperature-controlled, pathogen-free room accredited by the Association for Assessment and Accreditation of Laboratory Animal Care (AAALAC). Mice were provided *ad libitum* access to a chow diet (CD), high-fat diet (HFD), water, and 10% EtOH throughout the duration of the study. We provided both diets and liquids to determine if alcohol preference affected diet preference and vice versa. CD was a chow diet (5010; LabDiet, St. Louis, MO, USA) provided by the Comparative Medicine Research Unit staff and HFD (60% calories from fat) was purchased from Bio-Serv (S3282; Bio-Serv, Flemington, NJ, USA). Water was autoclaved and acidified, and 95% EtOH was diluted down to 10% with water and dispensed in standard mouse water bottles. Mice, diets, and liquids were weighed each week of the study. Water and 10% EtOH were rotated each week to minimize spatial bias (Figure 1A). Mice were euthanized at week 14 followed by exsanguination and tissue collection. Following euthanasia and tissue collection, we assessed blood phosphatidylethanol (PEth) to separate mice between alcohol-preferring (Pref, n = 4) and alcohol non-preferring (No Pref, n = 4) alongside alcohol consumption. One mouse was not included in the present study to provide an equal sample size. The separation of alcohol-preferring and non-preferring was used for group classification for all other downstream experiments [10]. We found that a threshold of >40 ng/mL for blood PEth levels also correlated with increased alcohol consumption in mice for the final four weeks (Figure 1B,C). While this is one of the first studies to measure blood PEth in mice, these threshold levels should be validated in other labs and animal models of alcohol consumption.

### 2.2. Glucose Tolerance Testing

At week 10, mice were subjected to a glucose tolerance test (GTT) as described previously [8].

### 2.3. Plasma Analyte Analyses

Blood collection was performed at euthanasia with heparinized needles. A total of 100 μL of whole blood was transferred into Lipid Panel Plus diskettes (Abaxis, Union City, CA, USA) to measure blood glucose and triglycerides on a Piccolo Xpress Chemistry Analyzer. Plasma endotoxin levels were examined by EndoLISA (BioVendor R&D, Asheville, NC, USA) measuring lipopolysaccharide (LPS). Plasma insulin, leptin, and glucagon were measured on the MesoScale Discovery (MSD) platform with the U-Plex Metabolic Group 1 (mouse) multiplex assay (Meso Scale Diagnostics, Rockville, MD, USA). PEth was measured in 50 μL of mouse whole blood dried on a blood card followed by organic solvent extraction as detailed in the Echelon Biosciences (Salt Lake City, UT, USA) PEth kit (K-5500Q).

### 2.4. Histological Analysis

Liver tissues were fixed in 10% neutral-buffered formalin for 48 h then transferred to 75% EtOH until tissue processing could begin. Formalin-fixed paraffin-embedded liver tissue were cut on a Leica Biosystems’ Histocore Autocut Automated Rotary Microtome at 5 μm. Hepatic morphology and qualitative steatosis assessment was performed by hematoxylin and eosin (Sigma Aldrich, St. Louis, MO, USA) (H&E) staining according to the manufacturer’s protocols.

### 2.5. Liver Tissue Analyte Analyses

Hepatic triglycerides were isolated and measured via a colorimetric assay (Ab65336; Cambridge, UK). Liver interleukin-10 (IL-10), interleukin-4 (IL-4), and tumor necrosis factor alpha (TNFα) were measured via ELISA on the MSD platform (Meso Scale Diagnostics, Rockville, MD, USA). Hepatic triglycerides and cytokines were normalized to total liver protein.

### 2.6. 16S Sample Preparation and Sequencing Analysis

Bacterial DNA isolated from mouse cecum was subjected to 16S amplicon sequencing as described previously [8]. The following microbiome diversity metrics were performed on 16S data including the Chao, Shannon, and Simpson indices. Data were assessed for normality and equal variance and subjected the proper statistical tests using STATom@ic [11]. Sequencing data are publicly available ENA under the following repository ID: PRJEB96050.

### 2.7. Whole-Organ mRNA Sequencing Analysis

Matched liver, ileum, white adipose tissue (WAT, visceral fat), skeletal muscle (quadricep), and brain tissue RNA was extracted from each group (n = 4) and analyzed by Agilent Tape Station analysis, and samples with RIN scores above 4 were used for library preparation and sequencing via the Illumina NovaSeq6000 instrument at 20 M reads per sample. Fastq files were processed with Trimmomatic and aligned to the Mus musculus genome (GRCm39) via STAR [12]. Normalized gene counts were generated via DeSeq2 [13]. Data were assessed for normality and equal variance and subjected to the proper statistical test using STATom@ic [11]. Sequencing data are publicly available ENA under the following repository ID: PRJEB89955. The number of shared DEGs across tissues were analyzed by creating a Venn diagram via molbiotools (https://molbiotools.com).

### 2.8. Statistical Analyses

Statistical comparisons were performed with GraphPad Prism (v.10.4.2) (La Jolla, CA, USA) using an unpaired Student’s *t*-test. Data are represented as the mean ± SEM and *p* < 0.05 was considered statistically significant.

## 3. Results

### 3.1. Alcohol-Preferring and Non-Preferring Mice Have Similar Liver Lipid Changes

Chiefly, we wanted to examine the metabolic and dyslipidemia impact in our mice that had access to all diets and liquids. Each cage housed two diets, HFD (60% kcal from fat) and chow diet, and two bottles: one of water and one of 10% EtOH (Figure 1A). Further, liquids were rotated weekly to avoid preference based on proximity to a preferred diet (e.g., avoidance of mice consuming more alcohol because of its location next to one diet or the other). Blood PEth levels were assessed to determine which mice had a long-term preference for alcohol (Figure 1B, Table S1). PEth levels can be detected for up to 4 weeks after cessation of alcohol consumption [14]. Thus, we used blood PEth as a biological marker of alcohol preference. This was consistent with the amount of EtOH consumed relative to water consumed for the final 4 weeks of the study (Figure 1C). We separated mice into “alcohol-preferring” mice and “non-preferring” mice based on blood PEth levels and alcohol consumption. There was no significant difference in body weight based on alcohol preference (Figure 1D). Blood triglycerides were lower with alcohol preference but not significantly (Figure 1E, Table S1). Plasma leptin levels were roughly 23% higher in alcohol-preferring mice; however, this was not a significant difference (Figure 1F). Liver triglycerides were slightly higher in alcohol-preferring mice, though not significantly (Figure 1G). Representative H&E-stained liver sections demonstrate similar levels of steatosis between alcohol-preferring and non-preferring mice (Figure 1H).

### 3.2. Alcohol-Preferring Mice Have Reduced Plasma Glucagon and Lower Fasted Blood Glucose Levels

**Figure 2:**
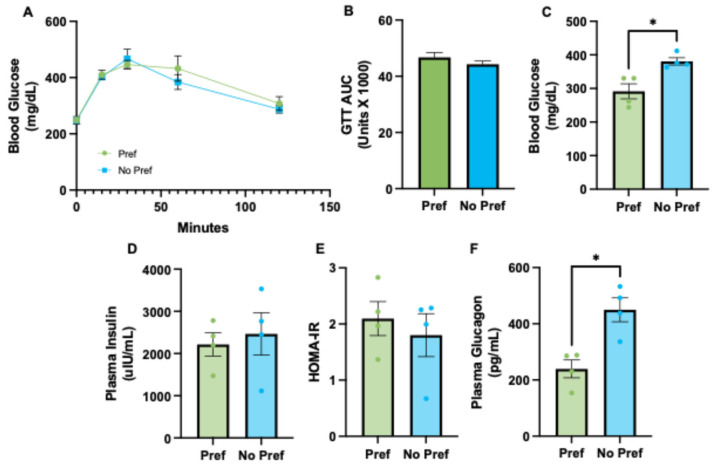
Alcohol-preferring mice demonstrate reduced plasma glucagon and fasting blood glucose. (**A**) Blood glucose levels (mg/dL) over the course of 120 min (GTT). (**B**) GTT area under the curve analysis. (**C**) Fasted blood glucose levels (mg/dL) at euthanasia. (**D**) Plasma insulin levels (uIU/mL) at euthanasia. (**E**) Calculated HOMA-IR scores. (**F**) Plasma glucagon levels (pg/mL) at euthanasia. An unpaired Student’s *t*-test was used to statistically compare our two groups where *p* < 0.05 was considered statistically significant and denoted by a *.

Next, we aimed to assess glucose homeostasis in alcohol-preferring vs. non-preferring mice. The glucose tolerance test (GTT) showed a similar response to glucose challenge between both groups (Figure 2A). The area under the curve (AUC) of the GTT results shows similar glucose tolerance between experimental groups (Figure 2B). It was observed that fasted blood glucose levels, following euthanasia, were significantly greater in alcohol non-preferring as compared to alcohol-preferring mice (Figure 2C, Table S1). No significant differences in plasma insulin levels (Figure 2D) or homeostatic model assessment of insulin resistance (HOMA-IR) (Figure 2E) were found. Curiously, HOMA-IR assessment was slightly higher in Pref mice. Plasma glucagon was significantly different and approximately 2-fold lower in alcohol-preferring mice (Figure 2F).

### 3.3. Microbiome Changes Associated with Alcohol Preference

**Figure 3:**
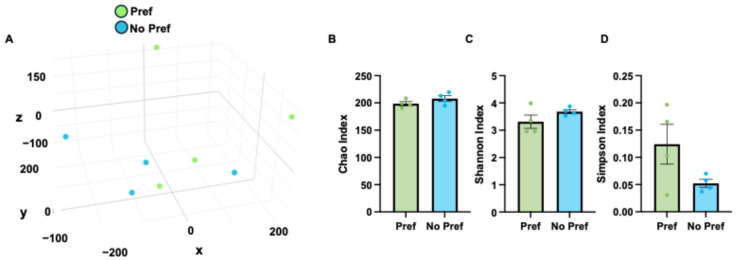
Microbiome changes associated with alcohol preference. (**A**) PCA of cecal 16S data. (**B**) Chao index of species richness for 16S data. (**C**) Shannon index of microbial diversity for 16S data. (**D**) Simpson diversity index for 16S data.

Next, we performed 16S amplicon sequencing of cecal contents to identify microbiome changes associated with alcohol preference. Principal component analysis demonstrates separation of alcohol-preferring vs. non-preferring mice based on 16S counts data (Figure 3A). Microbiome diversity was assessed via the Chao index (Figure 3B), Shannon index (Figure 3C), and Simpson index (Figure 3D). Across all indices, microbiome diversity was enhanced in alcohol non-preferring mice, although not significantly. In addition, there was a similar level of plasma endotoxin indicative of a similar level of gut barrier dysfunction with alcohol preference in this model (Figure S1).

### 3.4. Liver and Ileum Tissue Had the Greatest Number of Differentially Expressed Genes Associated with Alcohol Preference

**Figure 4:**
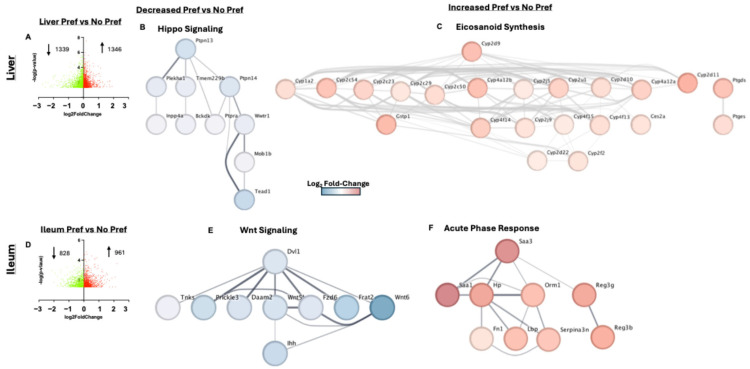
Liver and ileum tissue had the greatest number of differentially expressed genes associated with alcohol preference. (**A**) Volcano plot for significant liver DEGs between alcohol-preferring mice vs. non-preferring mice. (**B**) Hippo signaling gene cluster of downregulated hepatic genes in alcohol-preferring mice vs. non-preferring mice. (**C**) Eicosanoid synthesis gene cluster of upregulated hepatic genes in alcohol-preferring mice vs. non-preferring mice. (**D**) Volcano plot for significant ileum DEGs between alcohol-preferring mice vs. non-preferring mice. (**E**) Wnt signaling gene cluster of downregulated ileum genes in alcohol-preferring mice vs. non-preferring mice. (**F**) Acute-phase response gene cluster of upregulated ileum genes in alcohol-preferring mice vs. non-preferring mice.

To identify novel tissue-specific gene markers of alcohol preference, we performed whole-tissue RNASeq analysis of liver, ileum, brain, skeletal muscle, and white adipose tissue (WAT) (ranked from greatest to lowest number of DEGs). The liver and ileum are two primary target organs of alcohol toxicity [15] and, unsurprisingly, these tissues also had the largest amount of DEGs. Among the 1339 downregulated and 1346 upregulated genes in liver tissue in the volcano plot (Figure 4A), we identified several genes that play roles in metabolic dysfunction, inducing oxidative stress, and responding to or inducing inflammation. One of the top ten upregulated genes was Dusp6, which negatively regulates the MAPK/ERK signaling pathway via its phosphatase activity [16]. One downregulated gene ontology (GO) process in the liver included Hippo signaling (Figure 4B) which has been shown to impair liver regeneration [17]. Interestingly, eicosanoid synthesis was an elevated GO biological process in alcohol-preferring mice in the liver (Figure 4C). Eicosanoids are derived polyunsaturated fatty acids and can be pro-inflammatory or anti-inflammatory in nature depending on the lipid species [18]. Consistent with this finding, the hepatic pro-resolution cytokines IL-10 (Figure S2A) and IL-4 (Figure S2B) were lower in alcohol-preferring mice as compared to non-preferring, although not significantly. The top ten DEGs in the liver tissue for alcohol-preferring vs. non-preferring mice can be found in Table 1.

In the ileum, there were 828 downregulated and 961 upregulated DEGs associated with alcohol preference (Figure 4D). Among the top ten downregulated genes with alcohol preference in the ileum tissue was Wnt6, which is involved in the Wnt/Beta-catenin signaling pathway and gut epithelial wound healing [19]. Similarly, a network that is representative of the ileum genes downregulated with alcohol preference included Wnt signaling (Figure 4E). Interestingly, a network that was overrepresented by the upregulated ileum genes associated with alcohol preference included acute-phase response genes (Figure 4F). The top ten DEGs in the ileum tissue for alcohol-preferring vs. non-preferring mice can be found in Table 2.

### 3.5. Brain, Skeletal Muscle, and White Adipose Tissue Gene Markers of Alcohol Preference

**Figure 5:**
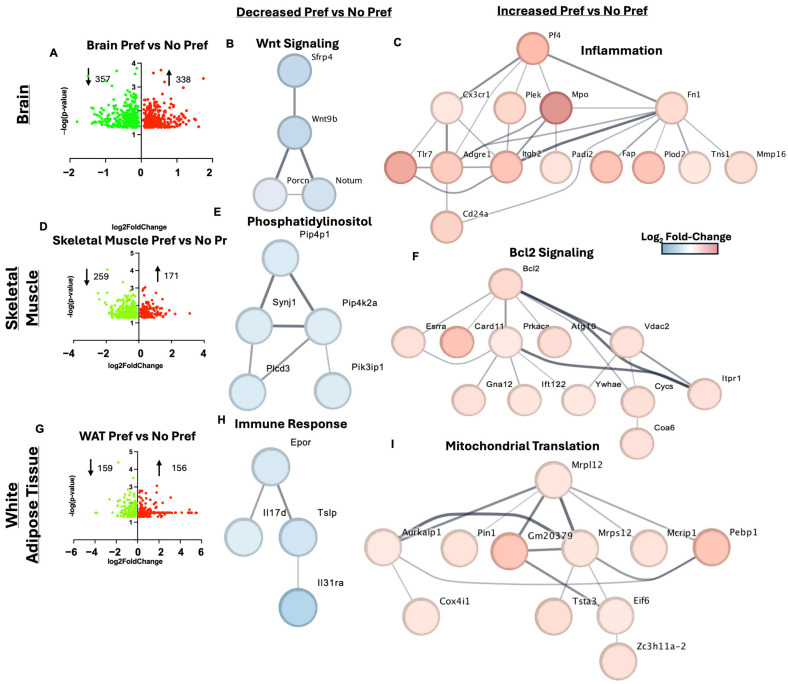
Brain, skeletal muscle, and white adipose tissue gene markers of alcohol preference. (**A**) Volcano plot for significant brain DEGs between alcohol-preferring mice vs. non-preferring mice. (**B**) Wnt signaling gene cluster of downregulated brain genes in alcohol-preferring mice vs. non-preferring mice. (**C**) Inflammation gene cluster of upregulated brain genes in alcohol-preferring mice vs. non-preferring mice. (**D**) Volcano plot for significant skeletal muscle DEGs between alcohol-preferring mice vs. non-preferring mice. (**E**) Phosphatidylinositol gene cluster of downregulated skeletal muscle genes in alcohol-preferring mice vs. non-preferring mice. (**F**) Bcl2 signaling gene cluster of upregulated skeletal muscle genes in alcohol-preferring mice vs. non-preferring mice. (**G**) Volcano plot for significant white adipose tissue (WAT) DEGs between alcohol-preferring mice vs. non-preferring mice. (**H**) Immune response gene cluster of downregulated WAT genes in alcohol-preferring mice vs. non-preferring mice. (**I**) Mitochondrial translation gene cluster of upregulated WAT genes in alcohol-preferring mice vs. non-preferring mice.

The brain had the third most DEGs associated with alcohol preference which included 357 downregulated and 338 upregulated genes (Figure 5A). One of the notable downregulated genes associated with alcohol preference in the brain was *Rbm3os*, also known as *2900002K06Rik*, which inhibits the Wnt/β-catenin signaling pathway [20]. Wnt signaling was a downregulated biological process in the brain with alcohol preference (Figure 5B). Genes involved in inflammation including *Mpo* (neutrophil marker) and *Adgre1* (macrophage marker) were upregulated with alcohol preference in the brain (Figure 5C). The top ten DEGs in the brain tissue for alcohol-preferring vs. non-preferring mice can be found in Table 3.

Skeletal muscle had the fourth most gene expression changes associated with alcohol preference including 259 downregulated and 171 upregulated DEGs (Figure 5D). *Prdm1* is involved in muscle cell differentiation [21] and was one of the top ten downregulated genes in the skeletal muscle of alcohol-preferring mice. Genes involved in phosphatidylinositol metabolism were downregulated with alcohol preference in the skeletal muscle (Figure 5E). *Bcl2* signaling was an upregulated process in the skeletal muscle with alcohol preference (Figure 5F). The top ten DEGs in the skeletal muscle tissue for alcohol-preferring vs. non-preferring mice can be found in Table 4.

WAT had the fewest DEGs associated with alcohol preference, including 159 downregulated and 156 upregulated DEGs (Figure 5G). One of the top ten downregulated genes with alcohol preference included *Gata4* which has been linked to alcohol use disorder (AUD) previously at the gene level [22]. Adipose tissue genes associated with the immune response including *Il17d* were downregulated with alcohol preference (Figure 5H). Mitochondrial translation was a GO process that was upregulated in adipose tissue in mice that preferred alcohol (Figure 5I). The top ten DEGs in the liver tissue for alcohol-preferring vs. non-preferring mice can be found in Table 5.

### 3.6. Shared DEGs Between Multiple Organs in Alcohol-Preferring Mice

**Figure 6:**
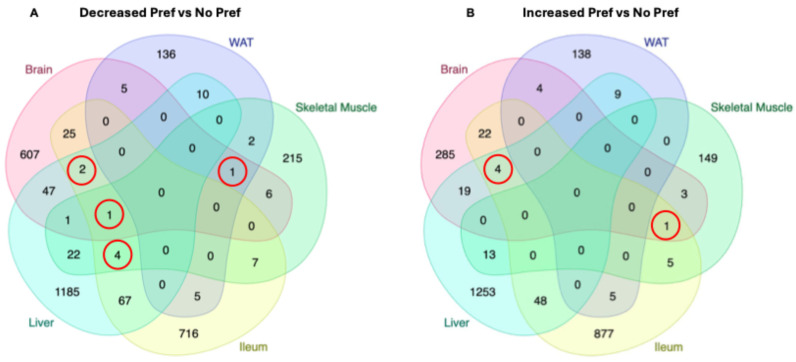
Shared DEGs between multiple organs in alcohol-preferring mice. (**A**) Venn diagram comparing the number of significant DEGs decreased with alcohol preference between liver, ileum, brain, skeletal muscle, and WAT. (**B**) Venn diagram comparing the number of significant DEGs increased with alcohol preference between liver, ileum, brain, skeletal muscle, and WAT. The red circles identifies the number of genes that are significantly upregulated with alcohol preference in 3 or more tissues.

Next, we aimed to identify gene expression changes that were shared between at least three organs. For genes that were downregulated with alcohol preference, we performed Venn diagram analysis (Figure 6A). *Ntf3* was found to be reduced with alcohol preference in brain, WAT, and skeletal muscle tissue. *Trim30d* was reduced in brain, skeletal muscle, and liver and *Gm47140* and *Trim11* were reduced in brain, ileum, and liver tissue with alcohol preference. Skeletal muscle, ileum, and liver had the greatest number of shared genes reduced with alcohol preference, which included *Sybu, Sowahc, Ophn1*, and *Hpgd*. The only gene reduced with alcohol preference in four organs (liver, ileum, brain, and skeletal muscle) was *Nek3*. We correlated blood PEth levels with plasma glucagon and liver, skeletal muscle, brain, and ileum tissue expression of *Nek3* (Figure S3). We found that glucagon levels are positively associated with PEth levels but negatively correlated with *Nek3* expression in the brain and skeletal muscle. In addition, we performed a Venn diagram analysis on genes that were increased in alcohol-preferring mice across the five tissues (Figure 6B). Four genes including *Cux1, Ptbp3, Gm15998*, and *Irf6* were increased with alcohol preference in the brain, ileum, and liver tissue. *Prune2* was a gene that had increased expression with alcohol preference in the brain, ileum, and skeletal muscle.

## 4. Discussion

Excessive alcohol intake with a hypercaloric diet is not only increasingly common, it is also underexplored in preclinical models. In this study, we used a MetALD mouse model to investigate the junction of AUD and MASLD—two factors commonly found in Western society. To categorize the groups based on long-term alcohol preference, we used PEth and weekly alcohol consumption relative to water consumption as indicators. PEth has a four-week detection window and has been used in humans to detect significant alcohol use [23]. However, there can be variation in the cut-off levels used to interpret PEth results across different clinical settings as well as inter-individual variability [10].

In the present study, we did not observe significant weight or lipid differences (e.g., blood cholesterol, liver triglycerides); however, we did find significant differences in circulating glucose and glucagon. As previously observed, chronic alcohol use tends to decrease endogenous glucagon levels, which reflects abnormal glycemic homeostasis [24]. Similarly, we identified significantly lower glucagon and glucose in alcohol-preferring mice, where insulin levels were similar in our two groups. These findings could suggest an alcohol-induced impairment of the maintenance of blood glucose levels in the fasted state, leading to hypoglycemia. In fact, EtOH consumption is associated with impaired gluconeogenesis and hypoglycemia in patients with alcohol-associated cirrhosis [25]. This may be a consistent finding in MetALD where glucagon production and gluconeogenesis are impaired with increased alcohol consumption.

We also observed, though not significant, that microbiome diversity was slightly higher in the non-preferring mice according to the Chao, Shannon, and Simpson indices. Like other studies across several species, chronic alcohol consumption frequently leads to lower microbiome diversity [26,27]. This phenomenon is usually accompanied by a higher deleterious bacterial species count and lower beneficial bacterial count. Piacentino et al. (2021) showed the reduction in beneficial bacteria and increase in potentially detrimental bacteria in alcohol-binge-drinking baboons, and increased lactobacillus genes in non-alcohol-preferring baboons and alcohol-dependent individuals after short-term abstinence [26]. Interestingly, we did not observe exacerbated endotoxemia in the alcohol-preferring mice. This may be due to the fact that both an HFD and alcohol consumption over 13 weeks lead to a similar level of gut barrier dysfunction independent of alcohol consumption alone.

The greatest number of DEGs were discovered in the liver and ileum tissues. Unsurprisingly, this finding is mainly because of the liver’s role as the primary alcohol metabolism site and ileal function in nutrient absorption and susceptibility to alcohol-induced barrier dysfunction and inflammation. As shown in Figure 4B, *Mob1b, Tead1,* and *Wwtr1* were downregulated genes in the liver that are directly associated with Hippo signaling. Downregulation of Hippo signaling could be highlighting liver regeneration impairment due to the pathway’s regulation of cellular proliferation, response to metabolic constraints, and regulation of the size of the liver. Other groups have found that dysregulation of the Hippo pathway altered the size of the liver promoting poor regeneration, hepatomegaly, or liver cancer [28]. One liver pathway associated with our upregulated gene set involved eicosanoid synthesis. Among several cytochrome P450s, the predominant gene is *Cyp2d9*, which is involved in metabolic and xenobiotic processes. *Cyp2d9* (human homolog *CYP2C9*) metabolizes arachidonic acid to eicosatrienoic acid epoxides [29]. Some eicosatrienoic acid epoxides are considered pro-inflammatory and play a role in the pathogenesis of ALD [30].

Recent studies have shown that proper Wnt signaling is required for liver protection against oxidative stress-induced apoptosis and intervention models have proven efficacious in attenuating ALD progression, indicating this pathway’s protective role in ALD [31]. Further, proper Wnt signaling is known to have critical homeostatic functions in the intestine [32]. The *Wnt6* gene is within the top 10 downregulated genes in the ileum, indicating that murine alcohol preference may have significantly impaired gut wound healing processes or contributed to deficiencies in the epithelial lining. Previous work has demonstrated that *Wnt6* is expressed highly in crypt epithelia, promoting progenitor cell function, and works in concert with Paneth cells to promote innate immunity and protection [33,34]. In the upregulated genes of the mouse ileum, we observed an elevation of several acute-phase response genes, where *Saa1* is in the top 10 upregulated DEGs. Acute-phase reactants (APR) are inflammation markers that exhibit significant changes in serum concentration during inflammation. Our pathway analysis displayed several connected APRs that were upregulated including *Saa1, Saa3, Hp, Orm1, Lbp*, and *Serpina3n* which are all positive APRs. The prevalence of positive ARPs in our dataset suggests that inflammatory events were more robust in alcohol-preferring mice [35]. The intestine can also generate an acute-phase response due to local inflammation [36]. Alcohol can directly damage the intestine leading to an inflammatory response [15] and contribute to increased gut permeability [37].

Interestingly, the brain had the third-highest amount of DEGs of the analyzed tissues and characterizing these DEGs and their affiliated pathways can possibly help us to understand AUD mechanisms and develop new treatments. One of the key reoccurring pathways of note is the Wnt signaling pathway, which was downregulated in brain tissue with alcohol preference, potentially attenuating neurogenesis [38]. Studies have shown that dysregulation of this pathway can have significant consequences for normal brain function [39]. Curiously, alcohol preference was associated with decreased Wnt signaling in both ileum and brain gene networks; however, the genes that were impacted differ (e.g., *Wnt6* vs. *Wnt9b*). Broadly speaking, these perturbations in Wnt signaling could negatively influence cellular differentiation, energy metabolism, and immune signaling. Specifically, altered Wnt signaling could contribute to organ pathology in these two locations by dysregulating the blood–brain barrier and intestinal epithelial lining, both promoting pathology [40,41]. Additionally, in the brain of alcohol-preferring mice, another upregulated gene set of inflammatory genes included *Pf4* (also known as *Cxcl4*), *Mpo, Cx3cr1, Plek*, and *Fn1*. *Mpo* was found in this overconnected network and in our top 10 upregulated brain DEGs. These findings suggest the prevalence of higher inflammatory pathway activation in our alcohol-preferring mice than the non-preferring mice. Recent work has demonstrated that not only does the overconsumption of alcohol cause direct deleterious effects, but chronic alcohol-induced inflammatory feedback cycles promote neurodegenerative and hepatic insufficiency pathologies [42,43]. Recent studies suggest a vicious cycle between alcohol-induced neuroinflammation as a driver for increased alcohol drinking patterns [44,45].

Further, our skeletal muscle tissue pathway analysis revealed several phosphatidylinositol-related genes that were downregulated with alcohol preference. The downregulation of these genes underlines how alcohol preference may disrupt broad signal transduction pathways and the maintenance of organelle membrane structures. It is well established that sarcopenia is a clinical characteristic of chronic alcohol consumption and current mechanisms attribute this feature to poor nutrition, impaired protein synthesis and enhanced degradation pathways, and chronic inflammation [46]. While more research is required to understand the specific genes that were downregulated in our dataset, previous work has alluded to disrupted phosphatidylinositol signaling being affiliated with alcohol-induced muscle function decline [47]. Interestingly, one study discovered a *PIP4K2A* polymorphism that was significantly associated with AUD over healthy controls [48]. Several genes were found to be upregulated in skeletal muscle with alcohol preference including the overconnected gene of interest, *Bcl2*. *Bcl2* is primarily a mitochondrial anti-apoptotic protein that sequesters apoptosis-promoting machinery [49]. Other genes of interest in this network included *Vdac2* [50] and *Itpr1* [51], which are critical to apoptosis signaling decisions. Given the upregulation of these genes, it may be that myocyte apoptosis is initiated due to overwhelming stress caused by alcohol but is prevented by BCL2, if functioning properly.

Of all tissues analyzed, WAT had the lowest number of DEGs, where we observed the downregulation of *Il17d* and *Il13ra* in alcohol-preferring mice. *Il17d* is a member of the IL17 family and one study found its deficiency to be associated with reduced weight loss [52]. Since our mice were fed an HFD, fat accumulation will be prevalent in tissues such as the liver and WAT, and *Il17d* downregulation may indicate less efficient lipid catabolism. *Il17d* has also been shown to inhibit hemopoiesis via IL-8 [53], where curiously we found *EpoR* (erythropoietin receptor) to be downregulated in this network and connected to *Il17d*. EpoR activation in adipose tissue stimulates lipolysis [54], and this paired with our findings suggest alcohol preference may contribute to the metabolic effects of MetALD. The upregulated genes in WAT due to alcohol preference included genes related to mitochondrial translation and regulation. *Mrpl12* and *Mrps12* are mitochondrial ribosomal encoding genes and *Cox4i1* is a subunit of cytochrome c oxidase. The upregulation of these genes may indicate increased protein turnover in response to heightened mitochondrial stress from chronic alcohol and HFD consumption. Previous studies have indicated that chronic alcohol consumption can promote insulin resistance and dysregulate adipokines [55]. The current study revealed higher plasma leptin in alcohol-preferring mice, although it was not significant.

We additionally examined genes that were differentially expressed in multiple tissue types to potentially act as biomarkers of preference or pharmaceutical targets for future studies. *Nek3* was significantly downregulated in all tissues except for WAT (undetected) and is a serine/threonine kinase involved in several cellular processes including the cell cycle, microtubule modulation, and migration [56]. Future studies are required to discern why *Nek3* is downregulated with alcohol preference in multiple tissues and what role if any it plays in alcohol addiction. *Ntf3* expression was also significantly downregulated in multiple tissues (brain, WAT, and skeletal muscle) and could be another potential marker of addiction. In addition, we found that brain, skeletal muscle, and ileum *Nek3* gene expression are negatively correlated with blood PEth and plasma glucagon levels. Currently, there has been no experimental connection between glucagon levels and tissue expression of *Nek3* in a model of alcohol preference. Future studies will determine the mechanism linking alcohol preference, glucagon, and *Nek3*. *Ntf3* is a protein-coding gene that is downregulated in HCC and associated with immune infiltration and T cell exhaustion [57]. In our study, *Ntf3* levels were significantly decreased in brain, WAT, and skeletal muscle tissues, highlighting its importance and connection to possible inflammation and cognitive decline. One study supports the idea of decreased *Ntf3* following alcohol exposure being involved in compensatory mechanisms of cognitive decline associated with AUD [58].

One upregulated gene across multiple tissues (brain, ileum, and liver) was *Cux1*, a transcription factor responsible for regulating gene expression, differentiation, and potentially the cell cycle [59]. No current studies have connected upregulated *Cux1* with alcohol preference. Potentially, CUX1 inhibition may reduce alcohol drinking behavior but more direct evidence is required to validate this preliminary finding. In addition, CUX1 has been associated with tumorigenesis in some cancers [60] and alcohol is considered a risk factor for multiple cancers [61]. Future studies should determine the interrelatedness between *Cux1* upregulation and the development of alcohol-induced tumorigenesis.

We observed *Irf6* to be significantly upregulated and shared between brain, ileum, and liver tissues. However, one study mentioned that in their model, Irf6 levels were significantly downregulated in HFD-induced steatosis and the overexpression of *Irf6* in hepatocytes attenuated lipid accumulation [62]. Our data shows that *Irf6* gene expression is upregulated in alcohol-preferring mice, which may reflect metabolic compensatory mechanisms to prevent disease progression. Future studies are required to delineate what function Irf6 is performing in MetALD and alcohol preference across multiple tissues.

While this study identified many new pathways and potential targets for alcohol preference in MetALD, there are also inherent limitations to this study. For one, only male mice were used, which may limit the translatability of these preliminary findings since sex is a critical factor in alcohol metabolism and AUD pathology. Future studies will explore alcohol preference in this MetALD model with female mice as well. Further, the sample size was adequate but small and will require further validation in a larger study. Many of the identified biomarkers require validation in gene KO or inhibitor/agonist studies to determine their effect on alcohol consumption in this MetALD model. The GO process analysis has inherent limitations and is associative in nature. Future studies are required to determine if the gene expression changes in respective tissues modify any of the biological processes identified in this preliminary study.

## 5. Conclusions

This study highlights the complexity between excessive alcohol intake, metabolic dysfunction, and molecular adaptations across multiple organs in a MetALD mouse model. Using this model, we characterized the metabolic phenotype and identified shared as well as tissue-specific transcriptomic changes stratified by alcohol preference. Some key findings including impaired glucose and glucagon homeostasis, reduced Hippo and Wnt signaling, greater inflammatory responses, and disruptions in lipid regulatory pathways. Genes like *Nek3*, *Ntf3*, *Cux1*, and *Irf6* emerged as potential biomarkers for alcohol preference in mice across multiple tissues. These findings reinforce the need to consider dietary influences when modeling AUD and suggest new molecular targets for future research in ALD. While this study and the associated findings are informative, they require further investigation due to the discussed limitations of the study.

## Figures and Tables

**Table 1:** Top ten liver DEGs.

Gene	*p*-Value	Log_2_ Fold-Change (Pref/No Pref)
*Cyp2a4*	0.0401689	−2.9088035
*Gm20274*	0.01709653	−2.7401112
*Cyp2c69*	0.04493801	−2.3655704
*Elf3*	0.02857143	−2.148375
*Ccnb2*	0.04900357	−2.0679971
*Col6a6*	0.03273774	1.66892196
*4931413K12Rik*	0.00385552	1.73018036
*Gm44620*	0.00917213	1.74749083
*Mup19*	0.00916766	2.05966658
*Gm14201*	0.03302515	2.1428187

**Table 2:** Top ten ileum DEGs.

Gene	*p*-Value	Log_2_ Fold-Change (Pref/No Pref)
*Hspe1-ps5*	0.00010337	−2.1375433
*Klhdc7b*	0.02068844	−1.9994922
*Cdh9*	0.0092713	−1.9256851
*Gm23123*	0.03234469	−1.8374187
*Gm43378*	0.0009537	−1.7702924
*Pcolce2*	0.02857143	1.51095014
*Sprr2a3*	0.02857143	1.71544386
*Saa3*	0.02162142	1.87438718
*Gm28875*	0.00886639	1.90822057
*Mrap*	0.00166207	2.1954666

**Table 3:** Top ten brain DEGs.

Gene	*p*-Value	Log_2_ Fold-Change (Pref/No Pref)
*FUT4*	0.00173059	−1.857677
*Gm23119*	0.02857143	−1.791453
*Gm42471*	0.00113454	−1.76197
*Fam24b*	0.01099322	−1.6992738
*Hsd3b7*	0.00034175	−1.4872414
*Kcng3*	0.04148866	1.70387877
*Tex15*	0.00042448	1.74945007
*Acsm3*	0.01424366	1.78181541
*Gm37212*	0.01554785	1.90608096
*H2ac6*	0.00935297	1.91243398

**Table 4:** Top ten skeletal muscle DEGs.

Gene	*p*-Value	Log_2_ Fold-Change (Pref/No Pref)
*Meis2*	0.00197744	−2.502308894
*Gm26642*	0.00453452	−2.430666649
*A430035B10Rik*	0.00668697	−2.068963567
*5430416N02Rik*	0.00906632	−2.008322262
*Tmem255a*	0.00183859	−1.943820645
*Zfp773*	0.0324688	1.62777072
*Gm45844*	0.02882195	1.655560946
*Adamts8*	0.01884012	1.86746702
*Gm43307*	0.03048931	2.225161348
*Slc4a1*	0.02857143	3.116616888

**Table 5:** Top ten white adipose tissue DEGs.

Gene	*p*-Value	Log_2_ Fold-Change (Pref/No Pref)
*Gata4*	0.02940105	−3.9475123
*Gm17455*	0.02940105	−3.8202673
*Gm25492*	0.00499012	−2.628718
*Aqp5*	0.0283722	−2.3473238
*Il31ra*	0.04392121	−1.9193447
*Samd5*	0.02940105	5.52984238
*Cdh17*	0.02107057	1.00 × 10^99^
*Clec18a*	0.02107057	1.00 × 10^99^
*Ttc39d*	0.02107057	1.00 × 10^99^
*Kndc1*	0.02107057	1.00 × 10^99^

## Data Availability

16S rRNA sequencing data are publicly available ENA under repository ID: PRJEB96050; mRNA sequencing data are publicly available ENA under repository ID: PRJEB89955.

## Supplementary Materials

The following supporting information can be downloaded at https://www.mdpi.com/article/10.3390/genes16101121/s1, Figure S1: Plasma endotoxin was not different with alcohol preference. Figure S2: Liver pro-resolution cytokines. Figure S3: Correlation between multiple analytes measured. Table S1: Blood measures for experimental mice.

## Abbreviations

The following abbreviations are used in this manuscript:

Alanine aminotransferase, ALT; aspartate aminotransferase, AST; alcohol use disorder, AUD; chow diet, CD; ethanol, EtOH; high-fat diet, HFD; metabolic dysfunction associated steatotic liver disease, MASLD; metabolic dysfunction and alcohol-associated liver disease, MetALD; steatotic liver disease, SLD.
