# Supplementary material for: Alcohol Preference Impacts Multi-Organ Transcriptome in MetALD
Source: Genes (Basel). 2025 Sep 23;16(10):1121. doi: 10.3390/genes16101121 (PMC12564771; doi:10.3390/genes16101121)
Supplement: Supplementary file 1 [file genes-16-01121-s001.zip › genes-3884959-supplementary.pdf]

Title: Alcohol preference impacts multi-organ transcriptome in MetALD
Supplemental Materials

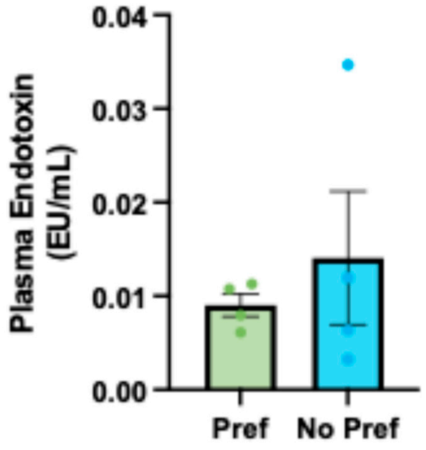

Supplemental Figure S1: Plasma endotoxin was not different with alcohol preference
Plasma endotoxin levels in experimental mice. An unpaired Student's *t*-test was used to statistically compare our two groups where a p< 0.05 was considered statistically significant and denoted by a *.

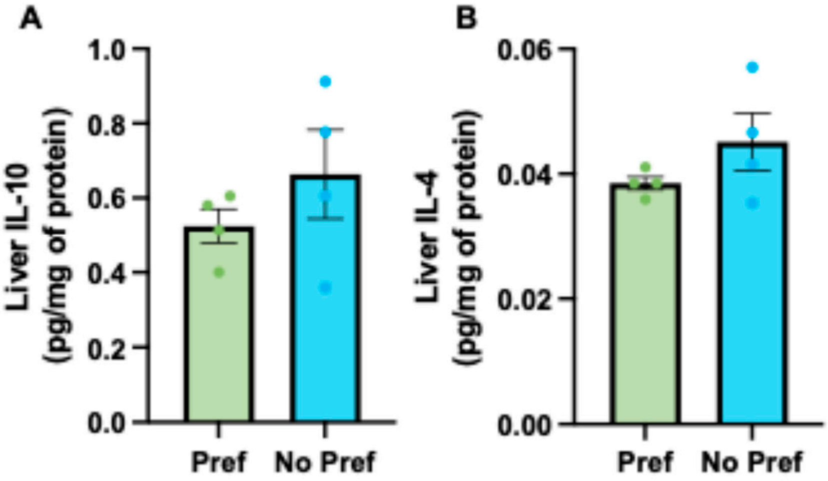

Supplemental Figure S2: Liver pro-resolution cytokines. A. Liver IL-10 levels. B. Liver IL-4 levels. An unpaired Student's *t*-test was used to statistically compare our two groups where a p< 0.05 was considered statistically significant and denoted by a *.

	PEth	Glucagon	Liver *Nek3*	Brain *Nek3*	Skm *Nek3*	Ileum *Nek3*
**PEth**	1.00	**0.65**	0.04	**-0.56**	-0.50	**-0.61**
**Glucagon**	**0.65**	1.00	0.07	**-0.61**	**-0.93**	-0.46
**Liver *Nek3***	0.04	0.07	1.00	0.00	-0.12	0.00
**Brain *Nek3***	**-0.56**	**-0.61**	0.00	1.00	0.44	**0.71**
**Skm *Nek3***	-0.50	**-0.93**	-0.12	0.44	1.00	0.22
**Ileum *Nek3***	**-0.61**	-0.46	0.00	**0.71**	0.22	1.00

Supplemental Figure S3: Correlation between multiple analytes measured. Correlation matrix for blood PEth levels, plasma glucagon levels, and liver, brain, skeletal muscle (Skm), and ileum *Nek3* expression. A simple linear regression analysis was performed and significant correlations ($p<0.05$) are denoted by being bold.

Table S1: Blood measures for experimental mice

	**Pref** (mean ± SEM)	**No Pref** (mean ± SEM)	**p-value**	**Fold-Change**
**Blood PEth (ng/mL)**	63.87 ± 6.0	33.24 ± 3.6	0.0048	1.92
**Blood Triglycerides (mg/dL)**	62.25 ± 3.3	72.00 ± 5.8	0.20	-1.16
**Blood Glucose (mg/dL)**	291.5 ± 22.5	380.8 ± 10.8	0.011	-1.31
